# Data Heterogeneity: The Enzyme to Catalyze Translational Bioinformatics?

**DOI:** 10.2196/18044

**Published:** 2020-08-12

**Authors:** Eli M Cahan, Purvesh Khatri

**Affiliations:** 1 Department of Medicine School of Medicine Stanford University Stanford, CA United States; 2 School of Medicine New York University New York, NY United States; 3 Department of Biomedical Data Sciences School of Medicine Stanford University Stanford, CA United States

**Keywords:** medical Informatics, health equity, health care disparities, population health, quality improvement, precision medicine

## Abstract

Up to 95% of novel interventions demonstrating significant effects at the bench fail to translate to the bedside. In recent years, the windfalls of “big data” have afforded investigators more substrate for research than ever before. However, issues with translation have persisted: although countless biomarkers for diagnostic and therapeutic targeting have been proposed, few of these generalize effectively. We assert that inadequate heterogeneity in datasets used for discovery and validation causes their nonrepresentativeness of the diversity observed in real-world patient populations. This nonrepresentativeness is contrasted with advantages rendered by the solicitation and utilization of data heterogeneity for multisystemic disease modeling. Accordingly, we propose the potential benefits of models premised on heterogeneity to promote the Institute for Healthcare Improvement’s Triple Aim. In an era of personalized medicine, these models can confer higher quality clinical care for individuals, increased access to effective care across all populations, and lower costs for the health care system.

## Background

Philosopher Karl Popper commented in 1934 that “non-reproducible single occurrences are of no significance to science” [1]. Yet, 85 years since this statement was made, science remains inundated with nonreproducible single occurrences. John Ioannidis famously wrote in 2005 that “most published research is false” [2]. Chalmers and Glasziou [3] later quantified the false positive rate of published science at 85%; the false positive rates in translational medicine may be even higher than this estimate. Up to 89% of studies demonstrating significant preclinical effects of novel molecules are nonreplicable [4], and the translation failure rate of novel interventions demonstrating significant effects preclinically that are never approved for clinical use reaches up to 95% [5]. These ranges may themselves be underestimates, since they are based on molecules assessed by pharmaceutical companies and in studies published in the highest-impact journals. The translation failure rate of less promising molecules is likely higher still.

In recent years, the emergence of multidimensional “big data” has endowed clinician investigators with more plentiful research substrate than ever before. However, issues with translation have persisted: despite innumerable statistically significant biomarkers identified in the preclinical setting, few of these generalize effectively. For example, 0% of proposed biomarkers for rheumatoid arthritis have demonstrated generalizability [6]. In addition, since enormous samples contribute sufficient statistical power capable of offsetting minute effect sizes, increasingly voluminous data may cause translation failure to become more rather than less of an endemic problem. Indeed, recent studies have noted a 36% deterioration of clinical effectiveness for molecules in Phase II trials [5].

We do not believe that the “depth” of samples (ie, cohort size) is responsible for the observed patterns in translation failure associated with big data. Rather, we believe that the problem is insufficient “breadth”; that is, the datasets used for discovery and validation fail to represent the diversity observed in distinctive real-world patient populations. In other words, by failing to represent the extent of real-world population diversity, we can define these datasets as inadequately *heterogeneous.*

There is already evidence for the effectiveness of translational bioinformatics premised on heterogeneity for conditions previously plagued by generalization failures, such as in the derivation of host response–based gene panels to predict sepsis and tuberculosis. These panels have outperformed all precedents developed without accounting for heterogeneity (including those using the most sophisticated machine-learning techniques); have been validated across time points, disease severity cohorts, and comorbidities; and have been generalizable across multiple continents [7-9].

In this paper, we highlight the tendency toward homogeneity in translational discovery and illuminate its negative implications. In contrast, we present heterogeneity as an ally rather than an enemy of meaningful translation. Finally, we describe the potential impact of incorporating heterogeneity into the process of translational bioinformatics for addressing the Institute for Healthcare Improvement’s Triple Aim: facilitating personalized medicine, alleviating a health care cost crisis, and resolving health disparities [10,11].

## Homogeneity Inherent to “Big” Translational Datasets

The core benefits of big data can be summarized in terms of volume (how much data are available), velocity (how quickly data are accumulated), and variety (how heterogenous the data are) [12]. Although the former two benefits have been harnessed extensively in translational research, the latter has not.

Datasets used for translational research may lack variety owing to three mechanisms: it may be absent, unevenly distributed, or inaccessible. The absence of variety results from constricted sourcing of data, leading to the funneling of homogenous features. One example is the exclusive use of healthy subjects for benchmarking, such as in immunocellular profiling for autoimmune disease [13]. The uneven distribution of variety within a dataset can lead to unintentional clustering of homogeneity, thereby filtering out heterogeneous characteristics. This is a digitized form of sampling bias: since heritability and penetrance both vary within populations, the findings in genome-wide association studies (GWAS) depend markedly upon the sampled cohorts [14]. Finally, variety may be present in the raw data but difficult to access, sequestering the heterogeneity due to technical hurdles. As dataset complexity increases, the risk of sequestration is amplified [15].

This becomes problematic in translational genomics, such as by producing “missing heritability” that is unexplainable from the processed dataset. It has been theorized that much of this “dark matter” (ie, the factors invisible in the processed dataset) relates to environmental influences. These environmental influences produce endophenotypes (expression profiles remaining latent until specific triggering exposures), which are epigenetic traits that can have strong contributions to phenotypic variation [16].

Homogenous datasets account poorly for differential environmental exposures and thus tend to be unreflective of transcriptomic diversity in broader populations. In turn, findings derived from such datasets may not extrapolate routinely beyond the experimental setting, thus precipitating translation failure.

## Homogeneity Rendered From “Big” Translational Datasets

Alternatively, homogeneity may be intentionally selected for within the dataset. The contemporary system of science is lubricated by two forms of currency—financial and academic—both of which present disincentives to embracing heterogeneity. On the one hand, budgetary constraints make inclusive, comprehensive methodologies (for instance, preclinical validation studies on multiple animal cohorts) either impractical or unaffordable [13]. On the other hand, the relentless pursuit of academic currency (reputation, garnered through publication) is more easily facilitated by exclusive, narrow methodologies. The inflation of effect sizes is readily conjured in well-controlled experimental populations subjected to investigator-dependent research methods [17].

This investigator-dependent variability—which produces what has been deemed the “vibration of effects”—fosters significant interstudy dissimilarity [17]. Investigator choices can fragment broad baseline populations into discrete clusters subjected to inconsistent exposures to create unbalanced terminal populations [7]. As Kaptchuk [18] pointed out:

Facts do not accumulate on the blank slates of researchers' minds and data simply do not speak for themselves…[the] evaluative process is never totally objective or completely independent of scientists’ convictions or theoretical apparatus.

Accordingly, one way to reframe the reproducibility crisis is as an *exclusivity* crisis. Intrinsic homogeneity (native to datasets) compounded by extrinsic homogeneity (rendered to datasets) yields a sort of “private epidemiology,” in which discrete study clusters are nonrepresentative of clinical diversity. This has been observed both in vitro and in vivo, where physiologic models poorly recapitulate real-world biology; up to 100% of findings based on observational data (such as vast catalogs of genetic signals) are not replicable [2,5,19]. Poor reproducibility has also been observed in silico, as predictive models premised on these limited feature sets have low external validity [12].

In short, the forces molding experimental homogeneity sculpt what become N-of-none studies. These are reflective of realities contained neatly within digital cells in spreadsheets rather than organic realities in patients.

## Heterogeneity in Translational Big Data: Today

More vivid depictions of organic (rather than spreadsheet) realities can be drawn from the introduction of heterogeneity to translational bioinformatics. Heterogeneity expands the analytical spectrum beyond the monochromatic shades of homogenous datasets to better represent real-world phenomena.

Just as meta-analyses mediate between-study biases in evaluation of treatment effects, the introduction of heterogeneity similarly allows for mediation of between-sample biases. Crucially, heterogeneity does not eliminate differences but rather synthesizes similarities [15]. The utility of heterogeneity comes from deriving commonality across diverse subgroups by including rather than excluding distinctive features.

This adheres to theories of systems biology (beyond Oslerian pathophysiology), which contextualize biological interactions in dynamic settings. Robust evidence has documented the inconsistent behavior of unique biological entities (ie, genomic, proteomic, and transcriptomic) “longitudinally” across time points and “latitudinally” across milieu [20]. Accordingly, cross-sectional studies in well-controlled samples seem to be ill-suited for explaining—much less, solving—polygenic diseases and polymechanistic syndromes.

Heterogeneity may be imputed experimentally by casting a wide net of investigators or of data samples. For the former, crowd-sourced collaboration has improved translational efforts compared with independent analyses across multiple indications (Table 1).

For the latter, construction of diverse datasets has yielded durable findings relevant for translation across numerous disorders previously plagued by false positives (Table 2). Protocols for introduction of heterogeneity by the use of multiple datasets are publicly available [21].

**Table 1:** Illustrative applications of crowd-sourced heterogeneity.

Title	Author	Year	Indication
Crowdsourced assessment of common genetic contribution to predicting anti-TNF treatment response in rheumatoid arthritis	Sieberts et al [22]	2016	Rheumatoid arthritis
Crowdsourced estimation of cognitive decline and resilience in Alzheimer’s disease	Allen et al [23]	2016	Alzheimer disease
Prediction of overall survival for patients with metastatic castration-resistant prostate cancer: development of a prognostic model through a crowdsourced challenge with open clinical trial data	Guinney et al [24]	2017	Prostate cancer
A community approach to mortality prediction in sepsis via gene expression analysis	Sweeney et al [25]	2018	Sepsis

**Table 2:** Illustrative applications of user-constructed heterogeneity.

Title	Author	Year	Indication
Leveraging heterogeneity across multiple datasets increases cell-mixture deconvolution accuracy and reduces biological and technical biases	Vallania et al [26]	2018	Autoimmune disease (systemic lupus erythematosus)
Identification of a common gene expression signature in dilated cardiomyopathy across independent microarray studies.	Barth et al [27]	2006	Cardiomyopathy
A common rejection module (CRM) for acute rejection across multiple organs identifies novel therapeutics for organ transplantation.	Khatri et al [28]	2013	Organ transplantation
Robust classification of bacterial and viral infections via integrated host gene expression diagnostics.	Sweeney et al [29]	2016	Upper respiratory infection
A community approach to mortality prediction in sepsis via gene expression analysis	Sweeney et al [24]	2018	Sepsis
Integrated, multi-cohort analysis identifies conserved transcriptional signatures across multiple respiratory viruses.	Andres-Terre et al [30]	2015	Influenza
Integrated multi-cohort transcriptional meta-analysis of neurodegenerative diseases	Li et al [31]	2014	Neurodegenerative disease
Integrated, multicohort analysis of systemic sclerosis identifies robust transcriptional signature of disease severity.	Lofgren et al [32]	2016	Systemic sclerosis
Genome-wide expression for diagnosis of pulmonary tuberculosis: a multicohort analysis	Sweeney et al [8]	2016	(Pulmonary) tuberculosis
Meta-analysis of continuous phenotypes identifies a gene signature that correlates with COPD disease status.	Scott et al [33]	2017	Chronic obstructive pulmonary disease (COPD)
A comprehensive time-course–based multicohort analysis of sepsis and sterile inflammation reveals a robust diagnostic gene set	Sweeney et al [34]	2016	Sepsis

Benefits to these strategies are exemplified by the studies mentioned in the Background section addressing tuberculosis and sepsis, respectively. The imputation of heterogeneity allowed for a 3-gene tuberculosis panel to be generalizable across 10 African countries [8,35] and an 11-gene panel capable of forming distinctive sepsis patient clusters to be validated in multiple nations [36]. Both of these panels, with their ability to accurately guide care for diverse patient groups (within and between populations), symbolize truly personalized medicine [7].

## Heterogeneity in Translational Big Data: Tomorrow

### General Prospects

Looking toward the future, the use of heterogeneity may play a prominent role in the advancement of translational bioinformatics by cultivating generalizability as a byproduct of representativeness.

**Figure 1:**
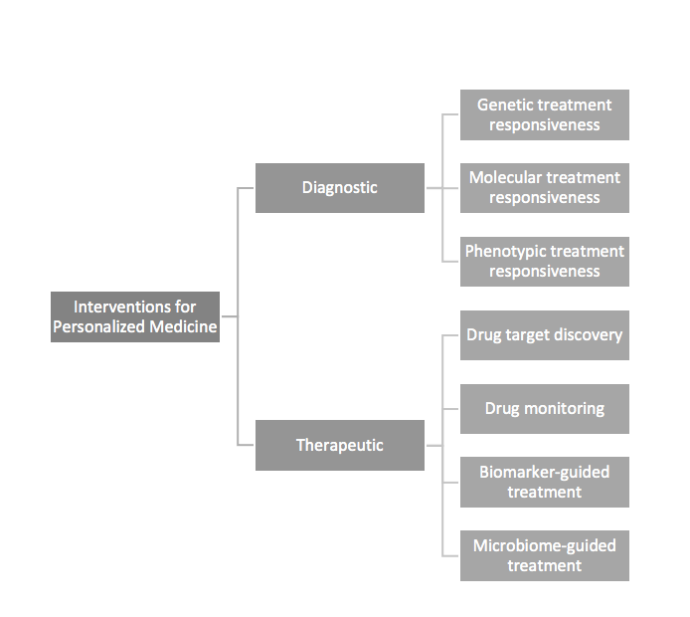
Modalities currently under investigation using translational bioinformatics to promote personalized medicine.

This bears substantial potential at the discovery stage, during which statistical significance is useful but not sufficient for predicting clinical effectiveness [2,19]. A myriad of diagnostic/prognostic and therapeutic modalities are being actively investigated for translation of personalized medicine, and validation will be crucial to distinguish the wheat from the chaff (Figure 1). Validation of novel diagnostics/prognostics (such as biomarkers) stands to benefit from heterogeneity given the aforementioned patient diversity across longitudinal and latitudinal scenarios [20]. Validation of novel therapeutics benefits from heterogeneity by enrichment of preclinical and clinical trials [37].

Establishment of data inclusiveness standards to supplement existing research guidelines (such as ARRIVE for preclinical studies and STROBE for observational studies) can accelerate the uptake of heterogeneity into best practices. The assimilation of heterogeneity into research practice in turn bears implications on personalized medicine, health care costs, and health disparities.

### Personalized Medicine

Leveraging heterogeneity in translational medicine may offer the quickest path to personalized medicine. It has been noted that increasing the number of datasets included in GWAS samples, controlling for sample size, markedly improves the predictive power of the obtained gene panels to a much greater extent than expanding the sample size alone [15].

This model also incorporates “dark matter” contributing to “missing heritability,” permitting the parsimonious identification of key biological pathways in spite of environmental differences between patient cohorts [16,38]. Moreover, observed differences may be informative rather than confounding: outliers bilaterally (such as weak or strong responders to interventions) are instructive and fertile sources for future investigation rather than “negligible.” N-of-one study becomes feasible within this paradigm.

Finally, while heterogeneity is not necessarily a panacea for discovery—studies utilizing heterogeneity to address acute respiratory distress syndrome have failed to find robust biomarkers—the utility of negative findings is bolstered by the methodology [39]. Evidence-of-absence investigations benefit greatly from additional rigor that more conclusively redirects researchers toward clinically meaningful prospects [13].

### Health Care Costs

Health care costs may be targeted from the sides of supply and demand alike. On the supply side, from the perspective of pharmaceutical companies, improved replicability of novel molecules reduces research and development costs devoted toward validation studies, which are currently estimated in the millions of dollars per agent tested [5]. Theoretically, this can allow for reduction in prices with preservation of profit margins. On the demand side, from the payor perspective, improved generalizability first enhances the cost-effectiveness of covered interventions, as clinical effects approach experimental effects [14]. Additionally, more reliable evidence-of-absence studies empower decision making for minimization of overutilized, misutilized, and ineffective interventions [13]. Finally, better understanding of “outlier” pathophysiology can promote the optimal management of “hot spotters”; that is, the oft-cited 1% of the population accounting for 33% of expenditures [40].

### Health Disparities

Reductions in payor costs, if passed on to consumers, improve the accessibility of health care. For example, the demonstration of predictive power for tuberculosis diagnosis using 3-gene rather than 71-gene panels implies marked reductions in testing costs (presuming proportional and consistent marginal costs). Furthermore, to the extent that technological barriers for 3-gene sequencing are lower, these diagnostics become available to populations outside of high-resource settings alone [8]. As long as more parsimonious models are adequately representative and maintain predictive power across population groups (as was the case in [8]), accuracy would be preserved in an equitable way while access is simultaneously enhanced.

Heterogeneity may also support the resolution of health disparities by virtue of inclusiveness. As previously discussed, multiplicity of sample sets benefits all populations, with disproportionately greater benefits for traditionally excluded populations [15]. In this way, channeling the “wisdom of crowds” refers not only to wisdom pulled by collaboration between investigators but also to the wisdom pushed by the comprehensiveness of study populations.

## Conclusion

In summary, we believe that research practices premised on sample homogeneity are important drivers of shortcomings in contemporary bench-to-bedside informatics. We assert that introduction of heterogeneity can favorably bend this trajectory. Uptake promoted by informal research culture change and formal inclusiveness criteria can lead to meaningful, sustainable, and equitable patient care in the future. In other words, the heterogeneity ethos echoes Osler’s original invocation for personalized medicine: “Just listen to the patient. He is telling you the diagnosis!”

## Abbreviations

GWAS: genome-wide association study
